# Elevated serum kynurenic acid in individuals with first-episode psychosis and insufficient response to antipsychotics

**DOI:** 10.1038/s41537-024-00483-z

**Published:** 2024-07-10

**Authors:** Alex Hatzimanolis, Stefania Foteli, Lida-Alkisti Xenaki, Mirjana Selakovic, Stefanos Dimitrakopoulos, Ilias Vlachos, Ioannis Kosteletos, Rigas-Filippos Soldatos, Maria Gazouli, Stylianos Chatzipanagiotou, Nikos Stefanis

**Affiliations:** 1 Department of Psychiatry, Medical School, National and Kapodistrian University of Athens, Eginition Hospital, Athens, Greece; 2 Neurobiology Research Institute, Theodore-Theohari Cozzika Foundation, Athens, Greece; 3 Department of Medical Biopathology, Medical School, National and Kapodistrian University of Athens, Eginition Hospital, Athens, Greece; 4 Department of Basic Medical Sciences, Laboratory of Biology, Medical School, National and Kapodistrian University of Athens, Athens, Greece; 5 World Federation of Societies of Biological Psychiatry, First Episode Psychosis Task Force, Barsbüttel, Germany

**Keywords:** Diseases, Psychosis

## Abstract

The tryptophan-metabolizing kynurenine pathway (KP) can be activated by enhanced inflammatory responses and has been implicated in the pathophysiology of schizophrenia. However, there is little evidence for KP dysregulation in the early course of psychotic illness. We aimed to investigate the potential immune-mediated hyperactivity of KP in individuals with first-episode psychosis (FEP) and the relationship with symptom severity and treatment response outcomes. Serum immunoassays were performed to measure peripheral levels of inflammatory cytokines (IL-1β, IL-10, TNF-a), KP rate-limiting enzymes (IDO/TDO), and kynurenic acid (KYNA) metabolite in 104 antipsychotic-naïve patients with FEP and 80 healthy controls (HC). The Positive and Negative Syndrome Scale (PANSS) and the Global Assessment of Functioning Scale (GAF) were administered to assess psychopathology and functioning status at admission and following 4-week treatment with antipsychotics. Cytokine and KP components levels were substantially increased in FEP patients compared to HC, before and after antipsychotic treatment. A significant positive correlation between pro-inflammatory IL-1β and KYNA levels was observed among FEP patients, but not in HC. Importantly, within-patient analysis revealed that those with higher baseline KYNA experienced more severe negative symptoms and poorer clinical improvement at follow-up. These findings suggest that KP is upregulated in early psychosis, likely through the induction of IL-1β-dependent pathways, and raised peripheral KYNA might represent a promising indicator of non-response to antipsychotic medication in patients with FEP.

## Introduction

The immune-inflammatory hypothesis of schizophrenia aetiology has attracted much interest as biochemical and genetic findings have implicated immune-related abnormalities in the development of psychosis^1,2^. Multiple lines of evidence indicate that low-grade inflammation could play a significant role in the pathophysiology of psychotic disorders, in particular schizophrenia^3^. An excessive inflammatory reaction in schizophrenia has been linked to infectious factors, autoimmune processes and stress-dependent activation of immune responses^1,4^. Substantial increases of peripheral levels of inflammatory markers, such as pro- and anti-inflammatory cytokines, have been documented in patients with psychosis-spectrum disorders during both the acute and chronic stages of the illness^5^. The activation of the immune system responses in psychotic disorders is further supported by recent evidence indicating an increased number of circulating immune cells, as well as elevated peripheral indexes of inflammation (i.e. neutrophil/lymphocyte ratio, C-reactive protein levels)^6–9^. Moreover, increased expression of immune-related genes has been observed in postmortem brain tissue from patients diagnosed with schizophrenia^10–12^, suggesting that aberrant neuronal immune gene activation could be part of schizophrenia pathophysiology^3^. The immune-inflammatory hypothesis for schizophrenia is further supported by findings demonstrating that antipsychotic medication might exert anti-inflammatory and immunoregulatory properties^13^.

Elevated peripheral levels of inflammatory cytokines and activated complement factors have been reported following a first-episode psychosis (FEP), suggesting that the activation of immune responses is already present in the early manifestation of psychotic symptoms^14–17^. Disturbances of immune-regulatory processes have been observed during FEP, including the over-activation of complement system components^17–19^, altered immune cell functions^20^, and differences in the absolute number of circulating immune cells^7,21^. In accordance with the above observations, more recent evidence has shown that the exacerbation of inflammatory responses might constitute a key pathogenic mechanism predisposing to psychosis onset, at least in a subgroup of individuals with FEP or schizophrenia characterized by higher inflammatory profile^2,22^. Similarly, observations for centrally increased levels of immune-inflammatory markers in cerebrospinal fluid (CSF) of patients with FEP denote the concurrent occurrence of enhanced neuroinflammatory processes in the brain^18,23^, possibly with detrimental effects on neuronal function^1,3^. Interestingly, it is hypothesized that disruptions of the complex interaction between peripheral immunity and the brain might represent a pathophysiological mechanism contributing to the etiology of schizophrenia^24^, which is also in agreement with indications for a possible impairment of the blood-brain-barrier (BBB) among individuals with FEP^23,25^.

It has been shown that inflammation and increased levels of pro-inflammatory cytokines in individuals with psychotic disorders could trigger the activation of tryptophan metabolism through the kynurenine pathway (KP), and the subsequent over-production of KP metabolite kynurenic acid (KYNA)^26–29^, which represents an endogenous allosteric antagonist of N-Methyl-D-Aspartate (NMDA) glutamate receptor in the brain^30,31^. Extensive experimental research has demonstrated that KP is physiologically regulated by enhanced immune responses, while KP metabolite fluctuations have been observed in major psychiatric disorders including schizophrenia, bipolar disorder, and major depressive disorder^32–34^. Higher levels of kynurenine (KYN) and KYNA metabolites have been detected in both blood and CSF from patients with schizophrenia^27,28,35–37^, as well as in white matter of dorsolateral prefrontal cortex postmortem samples^38^. The above observations provide evidence that the dysregulation of KP could potentially contribute to the development of psychotic symptomatology via molecular mechanisms negatively affecting glutamatergic neurotransmission^30,39^.

However, it is mentioned that fewer studies have noted reduced circulating KYNA levels among individuals with chronic schizophrenia or affective psychosis^40,41^, raising the possibility that KP regulation may be modified by confounding factors related to illness duration, symptom profile, and antipsychotic treatment. Moreover, meta-analytic findings have provided marginal evidence for decreased serum KYNA among acutely ill patients with psychosis-spectrum disorders who experience more severe clinical symptoms^42^. More recent investigations have implicated peripheral alterations of KP components to treatment-resistance in schizophrenia^43,44^, further suggesting the involvement of KP mediated glutamatergic abnormalities in the emergence of treatment resistance to antipsychotic medication^45^. In addition, it is intriguing that neurobiological findings link KYNA increase to impaired microglia-mediated synaptic pruning in schizophrenia^46^, likely implying that genetically-driven immune abnormalities associated with excessive synapse elimination (i.e. complement system activation)^12,47^ might promote KP metabolite variations that eventually potentiate synaptic loss.

With respect to psychosis, it is highly probable that KP dysregulation could originate from neuronal and/or peripheral immune-dependent aberrations that could result to disturbed glutamatergic neurotransmission^48,49^, and consequently to the presentation of positive, negative, and cognitive symptoms as imposed by the long-lasting glutamate hypothesis of schizophrenia^45,50^. As differences of KP components and their association with clinical outcomes have been minimally explored in the early stages of psychotic illness^51,52^, we sought to determine whether the peripheral levels of KP-regulating inflammatory cytokines, KP rate-limiting enzymes, and KYNA metabolite are up-regulated following FEP. Further, we investigated the relationship with symptom severity and response to antipsychotic medication.

## Methods

### Participants

All patients were enrolled in the Athens First-Episode Psychosis (FEP) Research Study, a collaborative research initiative on risk factors for psychosis development, involving five participating psychiatric clinics across the metropolitan area of Athens, Greece^17,53^. A total of 104 unrelated individuals (age range: 16–45 years, 72% males) diagnosed with FEP and never medicated with antipsychotics were included in the present study. Individuals with psychotic disorders due to another medical condition or acute intoxication, full-scale IQ < 70, developmental disorders, and kinship with an enrolled participant were excluded from the study. Clinician-based interviews were conducted by trained psychiatrists at admission to the study and following 4-week treatment with antipsychotics, using the Diagnostic Interview in Psychosis (DIP), a standardized semi-structured interview generating diagnoses according to different diagnostic algorithms on the basis of the Operational Criteria Checklist for Psychotic Illness (OPCRIT)^54^. Clinical diagnoses were established based on the International Classification of Diseases 10th Revision (ICD-10) diagnostic criteria^55^. The healthy control group (HC) comprised of 80 healthy volunteers (age range: 19–59 years; 74% males) with no history of psychiatric disorder, who donated blood samples for annual routine biochemical examination. Each volunteer underwent a brief medical interview from trained physicians to assess the presence of major mental illness and other neurological or immunological disorders. Written informed consent was obtained from every individual after a detailed description of the research objectives and the study protocol was approved by the ethics committee and the Institutional Review Board at Eginition University Hospital (Athens, Greece).

### Clinical assessments

Detailed sociodemographic and clinical information was gathered from every participant at inclusion. The Positive and Negative Syndrome Scale (PANSS)^56^, was administered from trained psychiatrists to evaluate the severity of psychopathology at baseline and at 4-week follow-up. PANSS total score, as well as positive, negative, and general symptom subscale scores were examined. Functioning status was assessed at both timepoints with the Global Assessment of Functioning (GAF) scale, which subjectively rates the social, occupational, and psychological functioning of an individual^57^.

### Evaluation of treatment response

Early response to treatment with antipsychotic medication was defined according to the consensus-based operational criteria for symptomatic remission, proposed by the Remission in Schizophrenia Working Group (RSWG)^58^. Due to the short-term follow-up assessment in our study design, the RSWG time criterion requiring at least 6 months of mild symptom severity was not considered in the definition of remission status. Following the above criteria, individual items of PANSS were assessed to classify FEP patients as remitters versus non-remitters to antipsychotic treatment at 4-week follow-up. In order to increase reliability with respect to treatment response evaluation, two additional dichotomous treatment outcomes were examined by classifying patient group based on (i) ≥50% reduction in PANSS total score from baseline to 4-week follow-up and (ii) GAF score ≥65 at follow-up, indicating good versus poor treatment response as per previous investigations in FEP patient groups^59–61^.

### Immunoassay measurements

Peripheral blood samples were routinely obtained from every individual (between 8.00 and 11.00 am) in BD Vacutainer SST Advance gel-containing tubes and centrifuged within 30 min (3000 rpm for 10 min) to collect serum samples for subsequent biochemical examination. Serum aliquots were kept frozen at −70 °C until further analysis. Serum concentrations of inflammatory cytokines (IL-1β, TNF-a, IL-10), KP rate-limiting enzymes (IDO/TDO) and kynurenic acid (KYNA) metabolite were determined using commercially available human-specific quantitative enzyme-linked immunosorbent assays: Interleukin-1 beta (IL-1β) ELISA Kit (detection limit: 0.40 pg/ml; BioVendor R&D Inc., Czech Republic), TNF-alpha ELISA Kit (detection limit: 2.30 pg/ml; BioVendor R&D Inc., Czech Republic), Interleukin-10 (IL-10) ELISA Kit (detection limit: 1.32 pg/ml; BioVendor R&D Inc., Czech Republic), Indoleamine-2,3-Dioxygenase (IDO) ELISA Kit (detection range: 0.82–200 ng/ml; Invitrogen, USA), Tryptophan 2,3-dioxygenase (TDO) ELISA Kit (detection range: 0.16–10 ng/ml; MyBioSource Inc., USA), Human Kynurenic Acid (KYNA) ELISA Kit (detection range: 2.47–200 ng/ml; MyBioSource Inc., USA). Measurements were performed in duplicate samples from each individual and the mean concentration value was calculated for downstream statistical analyses. Samples from the same individual with a difference between measurements >10% were excluded from the final dataset. To minimize the possibility of erroneous immunoassay-based KYNA quantification, an extensive comparison of mean KYNA concentration values in healthy individuals was conducted with prior investigations utilizing sensitive high-performance liquid chromatography and tandem mass spectrometry analytical protocols (LC/MS) to measure serum KYNA levels^62–68^. There was no evidence for notable discrepancies between the current and previous studies.

### Statistical analyses

Demographic characteristics were compared between FEP and healthy control (HC) groups using Pearson’s chi-squared test for categorical variables and Mann–Whitney *U*-test for continuous variables, as appropriate. For each cytokine and KP component, the differences in serum levels between study groups were examined using multiple regression models, adjusted for sex, age, smoking status, body mass index (BMI), and clinical site. Within the FEP group, serum measurements at baseline and after 4 weeks of antipsychotic treatment were compared by applying linear mixed-effects models (LMMs) to account for repeated measures, including time and BMI as random factors and adjusting for the same covariates as in the between-group analyses. Additional exploratory analyses were conducted by adding duration of untreated psychosis (DUP in weeks) and expert consensus diagnosis based on ICD-10 classification criteria (F20.0 vs. other) as potential confounding variables. Further, cytokine and KP components serum levels were separately examined in F20/non-F20 FEP groups to account for diagnosis-specific differences. Partial correlations between cytokine and KP components serum measurements in each study group, as well as associations between serum concentration of each component and PANSS subscale scores were examined by estimating Spearman’s rank correlation (rho) coefficients. Logistic regression models were performed to investigate whether baseline levels of cytokine and KP components could predict response to antipsychotic medication at follow-up. Individual regression models were applied for symptomatic remission, clinical improvement and functionality improvement outcomes, as previously described. For each response outcome, estimated odds ratio (OR), 95% confidence intervals (CIs) of the OR, and *p*-values derived based on the Wald statistic are reported. Serum concentrations for cytokines and KP components (average of duplicate measurements) were log_2_-transformed before analysis to ensure a normal distribution in both FEP and HC groups. Statistical tests were corrected for multiple comparisons using the Benjamini–Hochberg false discovery rate (FDR) method^69^, setting the cut-off for statistical significance at FDR-adjusted *p* < 0.05. All statistical analyses were performed using R version 4.2.2 package.

## Results

### Increased serum levels of inflammatory cytokines and KP components in FEP patients

**Fig. 1:**
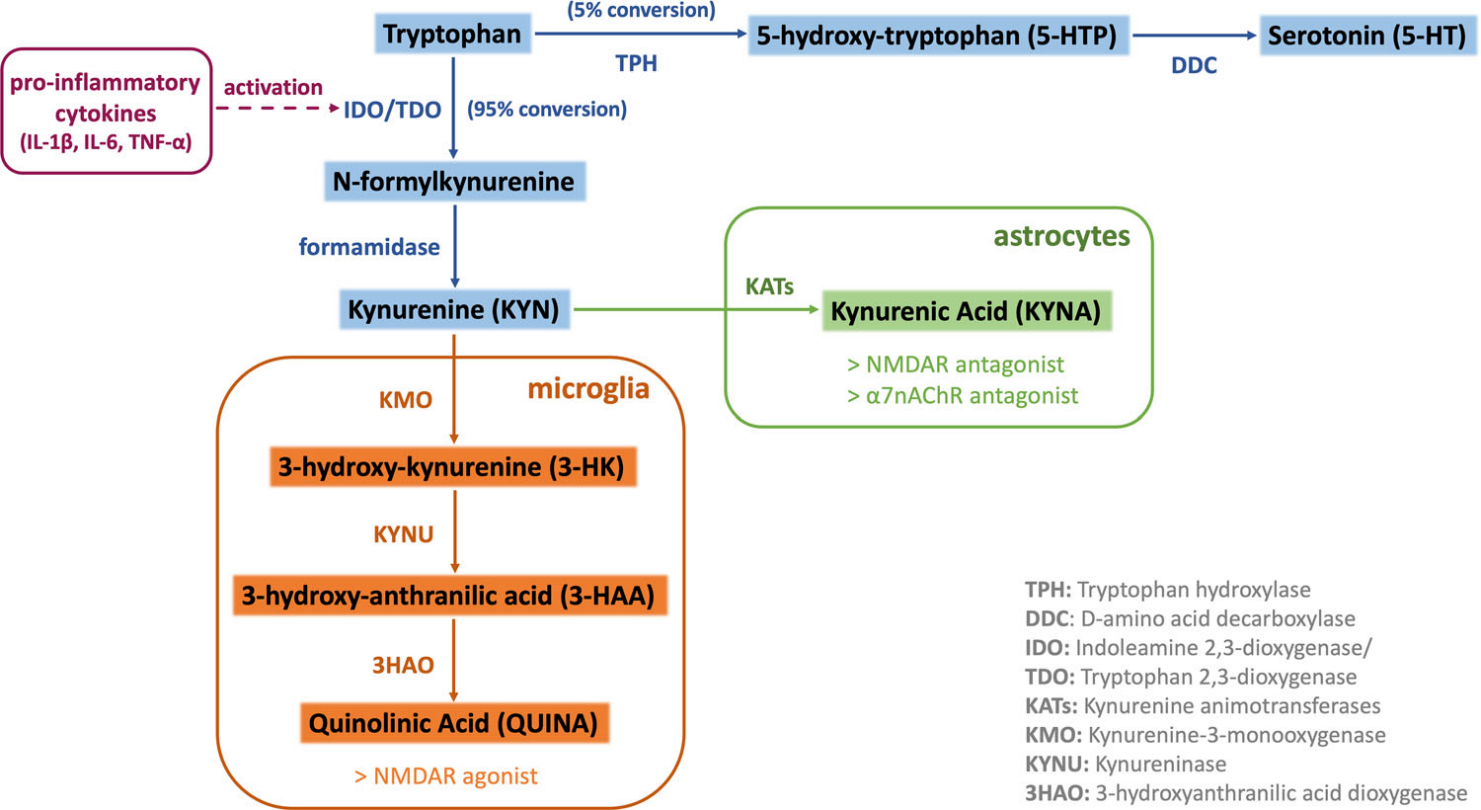
Schematic representation of the tryptophan-metabolizing kynurenine pathway. The key enzymes involved and main neuroactive metabolites are shown.

Demographic and clinical information of the study groups are presented in Table 1. Additional information on antipsychotic medication and mean dosages at discharge (4-weeks after the initiation of antipsychotic treatment) are provided in Supplementary Table S1. Initial analyses were conducted to evaluate the potential contribution of confounding variables on cytokine and KP components serum levels. In both groups, no significant differences were observed with regard to age, sex, smoking status, and BMI measurement. Nevertheless, all subsequent analyses were controlled for the above covariates to account for marginal confounding effects. Between-group comparisons revealed that peripheral levels of pro- (IL-1β, TNF-a) and anti-inflammatory (IL-10) cytokines, KP rate-limiting enzymes IDO/TDO, and KYNA metabolite were significantly higher in FEP patients compared to HC, at baseline and after 4-weeks of antipsychotic treatment administration (all FDR-adjusted *p* < 0.001). A greater than 2-fold increase was detected in the patient group for IL-10, TNF-a and IDO baseline levels, while a greater than 5-fold increase was noted for IL-1β, TDO, and KYNA baseline levels (Fig. 1, Supplementary Table S2). The concentrations of cytokines and KP components, at both baseline and follow-up measurements, did not differ in individuals who received a diagnosis of schizophrenia (54.5%), compared to the remaining individuals with FEP (Supplementary Table S3). Moreover, within-group analyses showed a minimal, yet significant, reduction in the concentration of pro-inflammatory cytokines and KP components among FEP patients following short-term treatment with atypical antipsychotics (IL-1β β = −0.24, *p* < 1e-06; TNF-a β = −0.15, *p* = 0.002; IDO β = −0.21, *p* < 1e-06; TDO β = −0.25, *p* < 1e-06; KYNA β = −0.08, *p* < 1e-04), except for anti-inflammatory IL-10 (β = 0.01, *p* = 0.520).

**Table 1:** Demographic and clinical characteristics of the study participants.

	First-Episode Psychosis (FEP)	Healthy Controls (HC)
Subjects (total N)	104	80
Sex (% males)	72	74
Age in years (Mean ± SD)	26.0 (7.0)	42.0 (10.5)
Current smoking (%)	59	51
BMI baseline (Mean kg/m^2^ ± SD)	22.7 (4.2)	24.8 (2.3)
BMI follow-up (Mean kg/m^2^ ± SD)	23.6 (4.3)	
Antipsychotic-naïve at baseline (%)	100	
DUP (Median in weeks)	12	
Schizophrenia (F20, %)	54.5	
Cannabis-related disorders (F12, %)	8.0	
Brief psychotic disorder (F23, %)	18.8	
Schizoaffective disorders (F25, %)	2.3	
Other psychotic disorder (F28, %)	3.1	
Manic episode/bipolar disorder (F30-31, %)	13.3	
Symptomatic remission at follow-up (%)	63	
>50% PANSS improvement at follow-up (%)	75	
GAF Baseline (Mean ± SD)	40.0 (14.5)	
GAF Follow-up (Mean ± SD)	59.5 (14.2)	
PANSS Baseline (Mean ± SD)		
Positive	28.2 (7.0)	
Negative	19.9 (9.6)	
General	49.6 (15.1)	
Total score	97.6 (25.7)	
PANSS Follow-up (Mean ± SD)		
Positive	13.4 (5.3)	
Negative	14.0 (7.0)	
General	29.8 (10.1)	
Total score	57.2 (19.5)	

*BMI* Body Mass Index, *DUP* Duration of Untreated Psychosis, *GAF* Global Functioning Scale, *PANSS* Positive and Negative Syndrome Scale.

### Correlations between cytokine and KP components serum levels

**Fig. 2:**
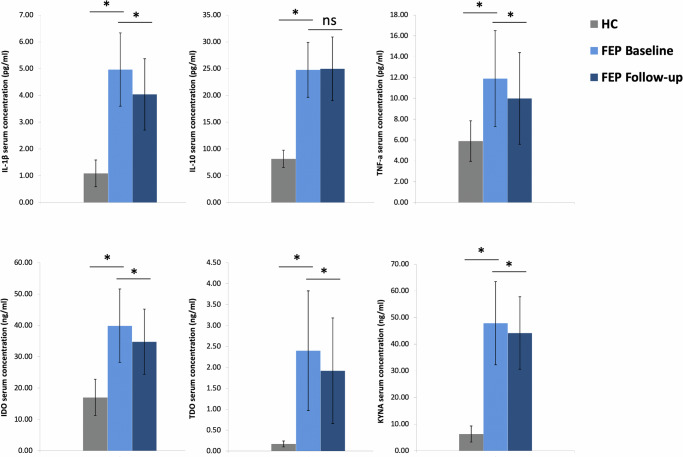
Comparison of inflammatory cytokine and kynurenine pathway (KP) components serum levels in patients with first-episode psychosis (FEP) and heathy controls (HC). Mean concentration values are shown at baseline and after 4-week treatment with antipsychotics. Error bars represent standard deviation (*FDR-adjusted *p* value < 0.05; ns non-significant).

The inter-relationships between cytokine and KP components serum levels were examined utilizing partial correlations, separately in HC and FEP study groups. Pro-inflammatory IL-1β levels were significantly positively correlated with KYNA at baseline and follow-up (rho=0.31, FDR-adjusted *p* = 0.043) in FEP patients, however a similar correlation was not observed in HC (rho = −0.03, uncorrected *p* = 0.952). Additionally, IDO and KYNA levels were positively correlated among FEP patients before treatment initiation (rho = 0.49, FDR-adjusted *p* = 0.03), and weakened after antipsychotic treatment (rho = 0.29, FDR-adjusted *p* = 0.168). No significant correlations between cytokines and KP components were detected in the HC group, except for a nominally significant negative correlation between IL-10 and TNF-a levels (rho = −0.29, uncorrected *p* = 0.01), which was not evident in the FEP group. Correlation coefficients for all tested comparisons between components in the HC and FEP groups are depicted in Fig. 2.

### Correlations with symptom severity and functionality status

**Fig. 3:**
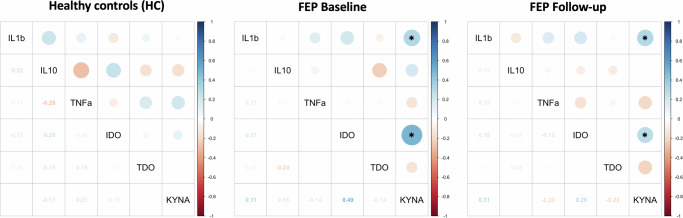
Pair-wise correlations between serum levels of inflammatory cytokines and KP components in healthy controls and individuals with first-episode psychosis (FEP). Statistically significant Spearman's rho correlations are indicated with an asterisk (*FDR-adjusted *p* value < 0.05).

In order to examine whether circulating levels of cytokine and KP components are associated with psychopathology severity and functionality among FEP patients, partial correlations with PANSS subscale scores and GAF total scores (baseline and follow-up assessments) were tested. Increased baseline KYNA levels were significantly associated with lower GAF score (rho = −0.33, FDR-adjusted *p* < 0.05) and more severe negative symptoms (rho = 0.29, FDR-adjusted *p* < 0.05) at follow-up (Table 2). In addition, we detected nominal associations between baseline KYNA and positive symptoms (rho = 0.25, uncorrected *p* = 0.017) as well as PANSS total score (rho = 0.24, uncorrected *p* = 0.023) at follow-up, which did not surpass correction for multiple comparisons. Likewise, given the observed positive correlation between baseline IDO and KYNA levels (Fig. 3), suggestive associations were also noted between higher IDO KP rate-limiting enzyme levels and PANSS positive subscale (rho = 0.26, uncorrected *p* = 0.006) and total scores (rho = 0.23, uncorrected *p* = 0.015), thus implying the up-regulation of KP in patients with FEP. Further exploratory analysis indicated that longer DUP and ICD-10 diagnosis of schizophrenia did not moderate the aforementioned significant associations with GAF or PANSS derived symptom severity scores. Nevertheless, a somewhat stronger correlation between KYNA and negative symptomatology was noted among patients with a schizophrenia diagnosis (rho = 0.23, uncorrected *p* = 0.04), compared to non-schizophrenia patients (rho = 0.05, uncorrected *p* = 0.788).

**Table 2:** Partial correlations between baseline cytokine and KP components serum concentration and severity of clinical symptoms and functionality, assessed at baseline and after 4 weeks of antipsychotic treatment.

Baseline assessment (drug-naïve state)						Follow-up assessment (4-weeks post-treatment)				
	PANSS Positive	PANSS Negative	PANSS General	PANSS Total	GAF Total	PANSS Positive	PANSS Negative	PANSS General	PANSS Total	GAF Total
**IL-1β**	−0.19	−0.17	−0.23	−0.27	0.18	0.12	0.05	0.09	0.09	−0.03
**IL-10**	0.14	0.23	0.17	0.21	0.03	0.09	0.19	0.17	0.16	−0.05
**TNF-α**	−0.05	−0.28	−0.10	−0.05	0.20	−0.07	0.21	0.05	−0.15	0.25
**IDO**	−0.08	0.06	−0.03	−0.07	0.09	0.26	0.16	0.20	0.23	0.11
**TDO**	0.01	0.01	0.16	0.13	0.08	−0.14	−0.07	−0.06	−0.07	0.05
**KYNA**	−0.19	0.14	−0.22	−0.15	−0.27	0.25	**0.29**	0.14	0.24	**−0.33**

Spearman’s rank correlation coefficients are indicated. Coefficients shown in bold denote statistical significance at FDR-adjusted *p* < 0.05

*PANSS* Positive and Negative Syndrome Scale, *GAF* Global Assessment of Functioning scale, *APs* antipsychotics.

Additionally, multiple regression models adjusting for baseline IL-1β levels were applied to examine whether the relationships between KYNA levels and clinical outcomes are mediated through pro-inflammatory IL-1β. There was no evidence for a reduction of the observed correlation coefficients, suggesting that it is unlikely the effect of KYNA on negative symptoms severity and functionality is attributed to increased IL-1β.

### Association between KYNA metabolite levels and treatment response

**Fig. 4:**
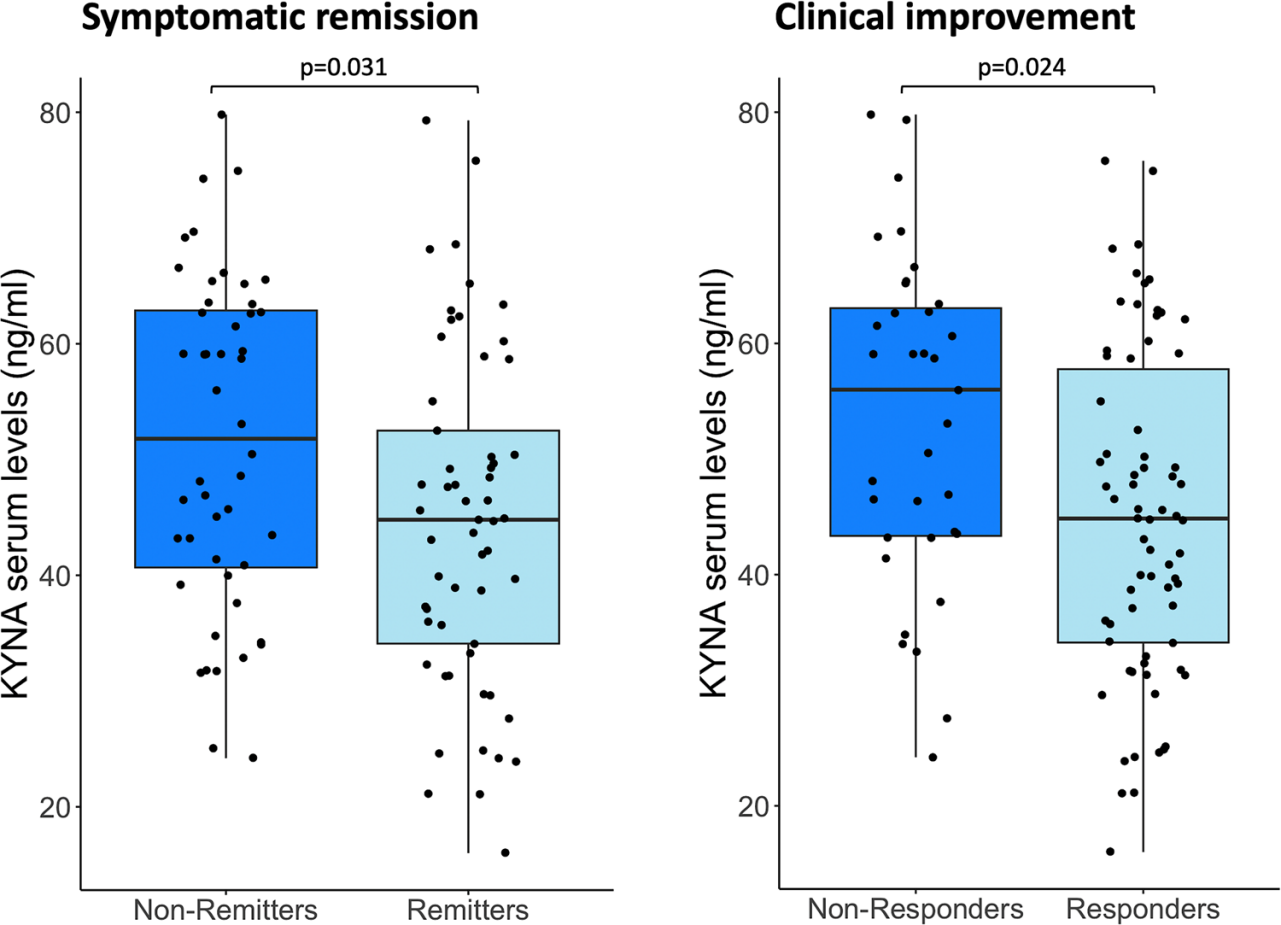
Association between serum kynurenic acid (KYNA) levels and treatment response. Box-plots depicting differences in mean serum KYNA concentration among FEP patients characterized as remitters/non-remitters following antipsychotic treatment based on the RSWG symptomatic criterion (left panel), and responders/non-responders based on the ≥50% PANSS reduction (right panel) at follow-up.

Extending our previous analyses, we sought to determine whether KYNA levels at baseline (i.e. antipsychotic-naive state) could be a significant predictor of short-term treatment response, over a 4-week treatment with antipsychotic medication. Based on the RSWG criteria, 55% of the patients achieved symptomatic remission at follow-up, and 67% of the patients were characterized as treatment responders according to the ≥50% PANSS total score reduction criterion (i.e., clinical improvement). Within FEP individuals, those with increasing KYNA concentration at admission (pre-treatment state) were more likely to exhibit poor symptomatic remission (remitters vs. non-remitters OR = 0.14, 95%CI: 0.03–0.50, FDR-adjusted *p* = 0.031), and inadequate clinical improvement at follow-up (responders vs. non-responders OR = 0.12, 95% CI 0.03–0.47, FDR-adjusted *p* = 0.024) (Fig. 4). Similarly, a trend association was observed between increasing baseline KYNA levels and reduced functionality among FEP patients at follow-up (41% with GAF score ≥65), implying unsatisfactory treatment response (OR = 0.19, 95% CI 0.04–0.71, FDR-adjusted *p* = 0.09) (Supplementary Table S4). There was no evidence for an association between baseline concentration of inflammatory cytokines or IDO/TDO enzyme levels and treatment response outcomes.

## Discussion

Multiple reports have demonstrated peripheral as well as brain KP metabolite differences in individuals with schizophrenia^28,33,35,36,40,42,70^, which might be induced by immune-inflammatory processes^24,32^. The findings of the current study support previous evidence that implicates KP dysregulation even at the early stages of psychosis^51,71,72^, and verify the increase of KYNA serum concentration in drug-naïve patients with FEP^73^. According to the results presented herein, raised inflammatory factors and more specifically IL-1β-dependent pro-inflammatory pathways likely activate KP in unmedicated patients with FEP. As the clinical diagnosis of schizophrenia may not be established during FEP, these results imply that the immune-related KP activation could not only pertain to schizophrenia, instead it might constitute an early hallmark of psychotic illness. Furthermore, this study suggests that the observed KP upregulation and the elevation of KYNA levels in acutely ill patients, is associated with intrinsic pathophysiological processes in early psychosis and it is not moderated by the exposure to antipsychotic medication. It is also noted that the short-term treatment with atypical antipsychotics is not sufficient to adequately normalize the observed exacerbation of pro-inflammatory markers and KP components. Yet, it remains to be seen in future studies whether the immune-mediated hyperactivity of KP persists over longer periods of antipsychotic treatment in newly diagnosed individuals with psychotic disorders.

In accordance with earlier findings^27,29^, our results suggest that the increase of pro-inflammatory cytokines in patients with psychosis triggers the over-activation of tryptophan metabolism through the induction of IDO rate-limiting enzyme activity and the subsequent increase of kynurenine metabolites, including neuroactive KYNA^31,70^. The above notion is further supported by previous reports showing the simultaneous increase of IL-1β and KYNA^35^, as well as the positive correlation between plasma IL-1β or IFN-γ and kynurenine (KYN) levels in patients with schizophrenia^40,72^. Similarly, the positive correlation between IL-1β and KYNA levels in the FEP group, at both baseline and follow-up measurements, most likely denotes an immune-mediated upregulation of KP in early psychosis. According to experimental evidence, inflammatory processes involving IL-1β-dependent cellular mechanisms might be responsible for the enhanced KYNA production via the stimulation or over-expression of tryptophan-catabolizing IDO/TDO enzymes^32^. Although no relationship was observed between IDO and IL-1β/TNF-a pro-inflammatory cytokine levels in this study, a positive correlation between IDO and pro-inflammatory IFN-γ was found in an independent FEP study^71^. Moreover, both the aforementioned and our study have detected increased IDO serum levels in unmedicated patients with FEP, which is an additional indication of KP hyperactivity and a plausible inflammation-related pathophysiological alteration linked to psychosis onset. Of note, a greater activity of IDO has also been observed in the brain and serum of schizophrenia patients^27,70^, suggesting that common molecular processes are responsible for the up-regulation of KP in the brain and blood.

It is well recognized that KYNA inhibits the function of N-methyl-D-aspartate (NMDA) receptor in the brain, acting as a non-competitive antagonist of the glycine-binding site^49^. Increased CSF KYNA levels have been detected in schizophrenia^28,36^, whereas similar observations in FEP are still lacking. It could be thus hypothesized that elevated KYNA levels in the brain of patients with psychotic disorders might exert a negative impact on glutamatergic neurotransmission, therefore exacerbating the severity of positive, and negative psychotic symptoms^49,50^. It is acknowledged that this study demonstrates a relationship between higher peripheral KYNA levels and greater severity of psychopathology among FEP patients, nevertheless we cannot infer that a similar increase of brain KYNA emerges during the early stages of psychosis, since circulating KYNA is essentially released by activated immune cells and is unable to infiltrate brain tissue unlike its precursor kynurenine^74^. It is thus possible that the excessive tryptophan metabolism in blood could result to the over-production of kynurenine, which crosses the blood-brain barrier and eventually is converted to KYNA by astrocytes in the brain^32,74^. It is of interest that growing evidence argues for a regular neuro-immune communication through the activation of KP and kynurenine metabolism, which could be deregulated in neurological and psychiatric disorders^28,32,74^. In line with the above notion, it has also been suggested that circulating regulatory T cells (Tregs) could contribute to impaired glial cell functioning in schizophrenia (i.e. astroglial over-activation, microglial-mediated synaptic pruning)^75^, and therefore to disrupted neuro-immune mechanisms that potentially result to central alterations of KP metabolites and dysregulated neurotransmission processes.

Several studies have shown higher CSF KYNA levels in schizophrenia^27,28,36,70,76^, whereas findings have been inconsistent with regard to serum or plasma^35,36,40,51^. The current study is the largest so far reporting increased serum KYNA levels among antipsychotic-naïve FEP patients. Prior investigations have reported either elevated^73^, or reduced^51^ peripheral KYNA in FEP patient groups compared to healthy individuals, although the latter studies included smaller number of patients with FEP. It is plausible that the variability of measured KYNA levels in different studies could be related to clinical factors associated with differential symptom profiles, psychiatric comorbidity, and medication effects between FEP patient groups. For example, the significantly shorter duration of illness before treatment initiation in the current study and the greater number of antipsychotic-naïve individuals could explain KP hyperactivity, compared to previous findings in FEP patients^51^. Given the considerable clinical heterogeneity among patients with FEP, it is argued that a range of clinical aspects and state-dependent symptom manifestations or psychological stress might be responsible for the observed KP metabolite disturbances during FEP or even at later stages of schizophrenia and bipolar disorder^38,77,78^. Consequently, additional research is required to fully elucidate the causes of KP dysregulation in psychotic disorders, and clarify whether this constitutes a pathological reaction or a physiological mechanism to essentially compensate the effects of enhanced inflammatory responses^79^.

This study provides novel evidence for an association between higher circulating levels of KYNA and poor early response to antipsychotic treatment among FEP patients, not previously exposed to antipsychotic medication. As of yet, the potential relationship between KP dysregulation in patients with psychotic disorders and antipsychotic treatment response in longitudinal studies is lacking. Additionally, no prior study has ever examined KP metabolites in relation to treatment outcomes in patients with FEP. Interestingly, a recent study found increased peripheral kynurenine (KYN) levels in patients with treatment-resistant schizophrenia (TRS) compared to non-TRS patients, and a positive correlation between KYNA and severity of psychopathology^43^. On the contrary, reduced KYN/KYNA levels were reported among TRS patients compared to healthy individuals in a different study^44^. Although the results of the above studies may be seen as preliminary, due to the limited number of cases examined, they suggest that KP activity could be differently modulated in non-responders to antipsychotic treatment or could be substantially influenced by clozapine prescription to TRS patients. However, it should be noted that the present study is restricted by the short follow-up assessment period and thus definite arguments about the involvement of KP alterations in treatment resistance cannot be postulated. Future prospective studies investigating treatment outcomes in larger groups of FEP patients over a longer follow-up evaluation will provide conclusive evidence for the predictive ability of KP metabolite changes in discriminating patients with TRS from the early clinical manifestations of psychosis.

A few limitations should be stressed with regard to the findings of this study. First, it is outlined that peripheral KP metabolite alterations may not reliably mirror brain levels in patients with FEP^70,76^, therefore no assumptions could be made for the impact of excessive serum KYNA on neuronal function. It remains to be verified whether a concordance exists between blood and brain KP metabolites in early psychosis, as the molecular mechanisms regulating kynurenine metabolism could considerably differ between blood and brain tissue. Second, this study is limited by the lower analytical accuracy of the immunoassay method utilized to measure serum KYNA concentration, which might have influenced association results. We note, however, that our measurements do not significantly deviate from the reported metabolite levels in previous studies using more accurate liquid chromatography analytical procedures. Third, the short-term follow-up assessment does not permit the evaluation of the anti-inflammatory action of antipsychotic treatment^13^, which could suppress peripheral KYNA levels over the course of the illness. Lastly, this study examined a heterogeneous population of FEP patients with both affective and non-affective psychotic disorders, who were followed-up for a limited time period that prohibited a firm diagnostic classification. Therefore, it is stressed that future studies with longer follow-up assessments are needed to delineate whether the impact of raised peripheral KYNA levels on antipsychotic non-response is restricted to patients with early-stage schizophrenia.

## Conclusion

Overall, the results of the present study support the immune-mediated upregulation of KP in antipsychotic-naïve individuals with psychotic disorders^80,81^, providing evidence for an increased tryptophan metabolism at the early phases of the illness via the induction of the KYNA producing branch of the KP. More importantly, the observation of higher KYNA levels among FEP patients with insufficient treatment response suggests that the over-activation of KP potentially triggers an imbalance of glutamatergic transmission through NMDA receptor hypofunction, which has previously been implicated in the development of TRS^82^, and has been associated with inadequate symptomatic remission in FEP following treatment with antipsychotics^83^. As the majority of patients with TRS do not respond to first-line antipsychotics from the onset of psychosis^84^, it is concluded that elevated peripheral KYNA concentration might prove a promising peripheral indicator of early non-response to antipsychotic treatment in individuals with FEP. Considering recent evidence that KYNA could stimulate synaptic pruning in schizophrenia and impair cerebral blood flow^46,85^, our findings support the pathophysiological role of higher KYNA in psychotic disorders and imply that potent modulators that impede KYNA over-production could hold promise for the development of novel and efficient therapeutic agents with antipsychotic properties.

### Supplementary information

Supplementary Tables

## Data Availability

Experimental and/or clinical data analyzed in this study is available from the corresponding author upon reasonable request.
