# Supplementary material for: Elevated serum kynurenic acid in individuals with first-episode psychosis and insufficient response to antipsychotics
Source: Schizophrenia (Heidelb). 2024 Jul 10;10(1):61. doi: 10.1038/s41537-024-00483-z (PMC11237022; doi:10.1038/s41537-024-00483-z)
Supplement: Supplementary file 1 — Supplementary Tables [file 41537_2024_483_MOESM1_ESM.docx]

**Supplementary Tables**

**Table S1.** Antipsychotic treatment information at discharge (4-week follow-up) in individuals with FEP (n=104)

| **Antipsychotic**  **medication** | **Individuals (n)** | **Frequency (%)** | **Mean antipsychotic**  **dosage (mg)** |
| --- | --- | --- | --- |
| Olanzapine | 46 | 44.2 | 17.6 |
| Risperidone | 37 | 35.6 | 5.4 |
| Aripiprazole | 10 | 9.6 | 18.5 |
| Clozapine | 4 | 3.8 | 75 |
| Quetiapine | 3 | 2.9 | 100 |
| Paliperidone | 2 | 1.9 | 79.5 |
| Amisulpride | 2 | 1.9 | 1000 |

**Table S2.** Mean values (+/- standard deviation) for cytokine and KP component serum measurements

in individuals with FEP (n=104) and healthy controls (n=80).

|  | **Healthy controls (HC)** | **Baseline**  **FEP** | **Follow-up**  **FEP** | **FEP Baseline vs. HC**  **(p-value)** | **FEP Baseline vs. Follow-up**  **(p-value)** |
| --- | --- | --- | --- | --- | --- |
| **IL-1β** (pg/ml) | 1.09 (0.48) | 4.97 (1.37) | 4.04 (1.32) | <0.001 | <0.001 |
| **IL-10** (pg/ml) | 8.17 (1.53) | 24.77 (5.13) | 24.98 (5.94) | <0.001 | 0.520 |
| **TNF-α** (ng/ml) | 5.85 (2.03) | 11.85 (4.65) | 9.99 (4.42) | <0.001 | 0.002 |
| **IDO** (ng/ml) | 17.04 (5.75) | 39.87 (11.71) | 34.79 (10.39) | <0.001 | <0.001 |
| **TDO** (ng/ml) | 0.19 (0.04) | 2.35 (1.43) | 1.92 (1.26) | <0.001 | <0.001 |
| **KYNA** (ng/ml) | 6.27 (3.00) | 47.85 (15.58) | 44.20 (13.60) | <0.001 | <0.001 |

**Table S3.** Comparison of mean concentration (± standard deviation) of measured cytokines and KP components between FEP individuals, following stratification by ICD-10 schizophrenia diagnosis (F20 vs. non-F20)

|  | **Non-Schizophrenia diagnosis**  **(ICD-10 non-F20, n=47)** | **Schizophrenia diagnosis**  **(ICD-10 F20, n=57)** | **β (p-value)** |
| --- | --- | --- | --- |
| **Baseline measurement** |  |  |  |
| IL-1β (pg/ml) | 5.19 (1.32) | 4.81 (1.39) | -0.135 (0.196) |
| IL-10 (pg/ml) | 24.72 (5.05) | 24.86 (5.16) | 0.013 (0.895) |
| TNF-α (ng/ml) | 11.83 (4.53) | 11.56 (4.86) | -0.028 (0.798) |
| IDO (ng/ml) | 39.76 (10.89) | 40.06 (12.24) | 0.013 (0.899) |
| TDO (ng/ml) | 2.40 (1.43) | 2.34 (1.44) | -0.022 (0.847) |
| KYNA (ng/ml) | 45.59 (17.53) | 49.41 (14.04) | 0.121 (0.244) |
| **Follow-up measurement** |  |  |  |
| IL-1β (pg/ml) | 4.13 (1.45) | 3.97 (1.23) | -0.062 (0.553) |
| IL-10 (pg/ml) | 24.77 (5.91) | 25.13 (5.27) | 0.032 (0.743) |
| TNF-α (ng/ml) | 10.10 (4.56) | 9.84 (4.24) | -0.026 (0.826) |
| IDO (ng/ml) | 35.77 (9.61) | 34.28 (10.80) | -0.071 (0.480) |
| TDO (ng/ml) | 1.90 (1.19) | 1.95 (1.30) | 0.021 (0.852) |
| KYNA (ng/ml) | 42.65 (15.44) | 45.23 (12.13) | 0.094 (0.368) |

**Table S4.** Logistic regression results for the association between baseline cytokine and KP components levels

and treatment response outcomes at 4-week follow-up.

|  | **Symptomatic Remission**  **(RSWG criteria)** | | **Clinical Improvement**  **(≥50% PANSS reduction)** | | **Functioning Improvement**  **(≥65 GAF score)** | |
| --- | --- | --- | --- | --- | --- | --- |
|  | **Odds Ratio (95% CI)** | **p-value** | **Odds Ratio (95% CI)** | **p-value** | **Odds Ratio (95% CI)** | **p-value** |
| **IL-1β** | 0.69 (0.13-3.70) | 0.668 | 1.27 (0.23-7.14) | 0.773 | 0.74 (0.13-4.17) | 0.729 |
| **IL-10** | 0.94 (0.86-1.03) | 0.156 | 0.97 (0.88-1.06) | 0.497 | 0.99 (0.90-1.08) | 0.770 |
| **TNF-α** | 1.11 (1.00-1.23) | 0.063 | 1.08 (0.97-1.20) | 0.166 | 1.05 (0.95-1.17) | 0.349 |
| **IDO** | 0.99 (0.96-1.03) | 0.632 | 0.98 (0.94-1.01) | 0.212 | 0.99 (0.05-1.02) | 0.376 |
| **TDO** | 1.12 (0.81-1.56) | 0.485 | 1.14 (0.82-1.59) | 0.436 | 1.09 (0.78-1.53) | 0.599 |
| **KYNA** | 0.14 (0.03-0.53) | **0.005*** | 0.12 (0.03-0.51) | **0.003*** | 0.19 (0.05-0.75) | 0.018 |

Uncorrected p-values are shown (*indicates significance at FDR corrected p<0.05)
